# Factors Affecting Workplace Bullying Among Intensive Care Unit Nurses and Their Needs for Improving Organizational Culture: A Mixed‐Methods Study

**DOI:** 10.1155/jonm/4843347

**Published:** 2026-02-03

**Authors:** Eunhye Kim, Sun Joo Jang, Yujeong Kim, Haeyoung Lee

**Affiliations:** ^1^ Department of Nursing, Seoul National University Hospital, Seoul, Republic of Korea, snuh.org; ^2^ College of Nursing & The Research Institute of Nursing Science, Seoul National University, Seoul 03080, Republic of Korea, snu.ac.kr; ^3^ College of Nursing, Research Institute of Nursing Innovation, Kyungpook National University, Daegu, Republic of Korea, knu.ac.kr; ^4^ Red Cross College of Nursing, Chung-Ang University, Seoul, Republic of Korea, cau.ac.kr

**Keywords:** narcissism, nurses, organizational culture, personality, workplace bullying

## Abstract

**Purpose:**

We identified factors influencing workplace bullying (WPB)—personality traits and organizational culture—among nurses working in adult ICUs at a tertiary hospital in South Korea. Additionally, supplementary questions and in‐depth interviews were used to explore nurses’ organizational culture improvement needs.

**Background:**

Nurses, under pressure to provide high‐quality care, are frequently exposed to WPB and experience higher peer bullying rates than other professions. As WPB increases turnover and negatively affects organizations, addressing this issue is imperative.

**Methods:**

This mixed‐methods study involved nurses working in intensive care units of a tertiary hospital. Quantitative self‐reported data were collected from 161 nurses between June 29 and July 14, 2022, covering pathological narcissism, perfectionism, dark personality traits, and WPB experiences. Qualitative in‐depth interviews were conducted with 21 nurses (10 with less than 5 years’ experience and 11 with 5 or more) and analyzed phenomenologically.

**Results:**

Multiple linear regression analysis revealed that WPB experiences were significantly influenced by positive organizational culture, pathological narcissism, and dark personality traits. Qualitative data analysis identified three key theme clusters: “discomfort between novice and experienced nurses,” “demand for strong sanctions against WPB,” and “appeal to improve human infrastructure and the work environment.”

**Conclusion:**

Lower levels of positive organizational culture, higher levels of pathological narcissism, and more pronounced dark personality traits were associated with more severe WPB experiences. Considering individual personality traits and the lack of a culture of mutual consideration and respect as causes of WPB, it is evident that both individual and organizational efforts are necessary to improve the situation.

**Implications for Nursing Management:**

Understanding the WPB challenges faced by novice and experienced nurses is crucial. Addressing their specific needs requires concrete and tailored interventions. As advocated by nurses, strong WPB eradication policies must be established, alongside improvements in working conditions to alleviate nurses’ workload.

## 1. Introduction

Hospitals increasingly emphasize the need for precision and expertise from their staff to improve patient safety and quality of care. This rising expectation has intensified the pressures placed on nurses, who must deliver high‐level services under challenging circumstances. These increasing demands make nurses more susceptible to workplace bullying (WPB), a common phenomenon in healthcare settings [1]. Nurses experience WPB more frequently than do professionals in other fields, and even compared with other healthcare professionals such as physicians [2–4].

The COVID‐19 pandemic resulted in unprecedented challenges for the nursing profession, leading to severe understaffing, heightened moral distress, and increased workload, all of which are established precursors to organizational conflict and violence [5–7]. Recent global data and systematic reviews confirm that WPB prevalence among nurses is a serious concern, particularly in the postpandemic era. The World Health Organization and International Council of Nurses highlight the pandemic’s profound impact on the global nursing workforce, noting significant increases in burnout, stress, and intent to leave the profession, which fuels instability and hostility in the workplace [8, 9]. A recent systematic review [10] analyzing violence in high‐risk settings reports a pooled prevalence of verbal violence as high as 57% and physical violence at 31% against healthcare professionals in intensive care units (ICUs) [5]. An extensive systematic review focusing specifically on violence against nurses during care corroborates these data [11], stressing that organizational issues and staff shortages continue to increase nurses’ vulnerability [5]. These latest findings confirm that WPB is not merely a persistent problem but one that has been exacerbated and structurally embedded within the postpandemic healthcare system, demanding urgent investigation.

WPB in nursing is defined as repeated, harmful actions directed at an individual, often manifesting as verbal abuse, work‐related harassment, and exclusion, thereby creating a toxic work environment [12, 13]. This bullying has significant physical and psychological consequences for nurses, including increased stress, anxiety, depression, and physical health issues such as hypertension and fatigue [11, 14, 15]. Additionally, WPB impacts job satisfaction and productivity and increases turnover rates, further destabilizing nursing organizations and patient care quality [14]. Inexperienced nurses often adopt passive or avoidant coping strategies, which perpetuate conflict and prolong bullying incidents [16]. Furthermore, new graduate nurses who experience bullying are more likely to become perpetrators later and perpetuate the harmful cycle, highlighting the importance of identifying WPB predictors [17].

Recent studies have also explored how narcissistic vulnerability and dark personality traits (e.g., narcissism, Machiavellianism, psychopathy) contribute to bullying behavior in the workplace [17, 18]. Mentalization—the ability to understand and interpret others’ mental states—is a psychological process that can influence how individuals perceive and respond to bullying situations [17].

In contemporary healthcare organizations characterized by increasing social narcissism, nurses may encounter colleagues, as well as patients and caregivers, exhibiting subclinical or pathological narcissistic traits [19]. While pathological personality traits, including narcissism, are generally less prevalent among healthcare professionals compared with the general population [20], Mortell [19] has called for increased awareness and strategies to mitigate the harm caused by narcissistic behaviors within nursing teams and organizations. Despite its importance, the role of personality in nursing contexts remains underexplored [20].

ICUs represent high‐stakes, high‐acuity clinical settings characterized by critical patient care, frequent life‐or‐death decision‐making, and time‐sensitive multidisciplinary teamwork. Errors carry potentially serious consequences, and patient conditions may change rapidly—a context that demands constant vigilance and precision [10, 18]. This high level of responsibility, coupled with the fast‐paced nature of ICU work, creates intense stress and pressure, which can lead to tension, conflict, and power imbalances within the team. These factors, combined with hierarchical structures and low tolerance for error, make ICU settings uniquely susceptible to WPB. In fact, recent studies consistently report a higher prevalence of WPB in high‐risk departments such as the ICU compared with general wards [10, 21]. Therefore, the selection of ICU nurses as the primary study population is strongly justified for exploring WPB occurrence and the role of personality factors.

Against this background, we aimed to explore the relationship between nurses’ personality traits, organizational culture, and WPB in ICU settings. Using a mixed‐methods approach, we investigated how ICU nurses’ interactions, organizational culture, and workplace dynamics influence the prevalence of WPB. In addition, we identified specific predictors of WPB, including personality traits such as narcissistic vulnerability and dark triad traits, and examined the impact of ICU‐specific stressors on bullying behaviors. The findings may provide actionable insights to develop practical strategies for improving organizational culture, preventing WPB, and fostering healthier work environments in ICU settings.

## 2. Methods

### 2.1. Study Design

A mixed‐methods approach was utilized in this study, integrating both qualitative and quantitative data.

### 2.2. Participants and Data Collection

#### 2.2.1. Quantitative Study

The inclusion and exclusion criteria for participants in the quantitative study were as follows. Inclusion criteria are (1) nurses currently working in adult ICUs at a tertiary hospital in South Korea and (2) nurses providing direct patient care. Exclusion criteria are (1) nurses responsible for education or administrative duties who do not provide direct patient care, (2) unit manager, and (3) nurses with less than 3 months’ experience who are currently within the probationary period.

Sample size was determined using the G∗Power 3.1.9.7 program. For a multiple regression analysis with a two‐tailed test, 14 predictors, medium effect size (*f* ^2^ = 0.15), significance level of 0.05, and power of 0.80, the minimum required sample size was 136 participants. Considering a potential dropout rate of 15%, the target sample size was set to 160. To ensure objectivity in data collection, the survey was conducted by an external professional organization called Gallup Korea. Recruitment announcements were not posted publicly; instead, an informational guide and a survey link were sent to all eligible nurses via the hospital’s internal messaging system. Participation in the survey was entirely voluntary, and measures were implemented to prevent duplicate responses by restricting multiple submissions from the same IP address. The survey was conducted from June 29 to July 14, 2022, and data were collected from a total of 161 participants.

#### 2.2.2. Qualitative Study

For this qualitative study, we selected the participants from 161 individuals who had voluntarily agreed to take part in in‐depth interviews. According to Bartholomew et al. [22], phenomenological nursing studies with 5–25 participants generally achieve adequate quality, which is suitable for obtaining detailed descriptions of participants’ experiences and reaching data saturation. To explore the experiences of nurses with both extensive and limited experience, we held individual in‐depth interviews separately for nurses with less than 5 years’ clinical experience and those with 5 or more. We conducted the interviews until reaching theoretical saturation, where no new essential meanings emerged from the statements. A total of 21 nurses participated in the in‐depth interviews, with 10 having less than 5 years’ clinical experience and 11 having 5 or more.

We collected qualitative data from August 16 to September 7, 2022. Among the 161 participants who completed the quantitative survey, we selected those who had expressed willingness to take part in the qualitative study and who provided consent for in‐depth interviews, which were conducted by a professional interviewer. We informed the participants of the study’s purpose and method, including the recording of the interview content, and we obtained written consent from those who ultimately agreed to participate. The interviews were conducted in the Gallup Korea counseling room or at locations preferred by the participants, such as cafes near their workplaces or homes. Each participant took part in a single interview session, which lasted approximately 30–60 min. The interview questions were designed by a panel consisting of three professors with experience in WPB research and one nurse manager from a tertiary hospital. Key questions for the interviews included are as follows:

• How would you describe your relationships with colleagues and novice/experienced nurses in your current workplace?
• Have you experienced any difficulties in these relationships?
• Please describe your experience as a victim (or perpetrator) of WPB while working in the ICU.
• What are the challenges of working with other nursing colleagues?
• What do you think are the reasons for WPB?
• What do you think are the strengths and areas for improvement in the hospital’s education and support system for preventing and managing WPB?
• What measures do you think should be implemented to eradicate WPB?

With the participants’ consent, the professional interviewer recorded the interviews and transcribed them verbatim immediately after each session. Interviews started with neutral, open‐ended questions about workplace relationships and then proceeded to WPB‐related questions. Although formal member checking was not conducted, we preserved participants’ original wording by using verbatim, scenario‐like transcripts as the primary analytic material.

### 2.3. Measurements

Referring to a previous study [18], we collected the participants’ demographic traits and work‐related characteristics using a self‐report questionnaire. We measured pathological narcissism, perfectionism, dark personality, organizational culture, and WPB experience using the following instruments after obtaining permission from the original developers and the authors of the Korean versions.

#### 2.3.1. Pathological Narcissism

We used the Korean version [23] of the Pathological Narcissism Inventory [24], comprising 35 items to assess pathological narcissism. Each item is rated on a 7‐point Likert scale (scoring range: 0–210), where a higher score indicates more severe pathological narcissism. The Cronbach’s α was 0.95 (0.78–0.93) at the time of development [24], 0.92 (0.85–0.92) in the Korean version [23], and 0.95 in the present study.

#### 2.3.2. Perfectionism

We used the Korean version [25] of the Perfectionistic Self‐Presentation and Psychological Distress scale [26] to measure perfectionism. The original tool consists of three domains (perfectionistic self‐promotion, nondisplay of imperfection, and nondisclosure of imperfection) with 27 items. Each item is rated on a 7‐point Likert scale (1: never; 7: always). Eight items were removed during the process of cultural adaptation, and so the Korean version contains 19 items. The Cronbach’s α was 0.91–0.95 at the time of development [26], 0.85 (0.75–0.88) in the Korean version [25], and 0.87 in the current study.

#### 2.3.3. Dark Personality

We measured dark personality using the Korean version [27] of the 27‐item Short Dark Triad (narcissism, Machiavellianism, and psychopathy) [28]. Ten items were removed from the Korean version, and Machiavellianism and psychopathy were merged into a single factor for a total of two factors (narcissism and Machiavellianism–psychopathy) and 17 items. Each item is rated on a 5‐point Likert‐type scale (1: strongly disagree; 5: strongly agree), with the total score ranging from 17 to 85. The Cronbach’s α was 0.73–0.78 at the time of development, 0.85 (0.75–0.84) in the Korean version, and 0.85 in the present study.

#### 2.3.4. Organizational Culture

We assessed organizational culture using the Positive Nursing Organizational Culture Measurement Tool [29]. This 26‐item tool consists of four factors (positive leadership of the nursing unit manager, pursuit of common values, formation of an organizational relationship based on trust, and a fair management system). Each item is rated on a 5‐point Likert scale (1: strongly disagree; 5: strongly agree), and the total score ranges from 24 to 120. A higher score indicates a more positive perception of nursing organizational culture. This instrument was developed by Korean researchers for general nursing organizational contexts (i.e., not ICU‐specific), and its reliability and validity have been tested among hospital nurses in Korea [29]. The Cronbach’s α was 0.95 at the time of development and 0.95 in the present study.

#### 2.3.5. WPB Experience

We assessed WPB experience using the Korean version [30] of the Negative Acts Questionnaire–Revised (NAQ‐R) [31]. It comprises 22 items, rated on a 5‐point Likert scale, with scores ranging from 22 to 110. The Cronbach’s α was 0.93 at the time of development [31], 0.93 in the Korean version [30], and 0.95 in the current study.

### 2.4. Ethical Considerations

The study was conducted after obtaining approval from the Institutional Review Board (IRB) of Seoul National University Hospital (Approval No. H‐2205‐106‐1325, initial approval date: June 24, 2022). Considering the vulnerability of the participants and to ensure the objectivity of the survey data collection, the survey was outsourced to an external professional organization, Gallup Korea. A survey link, along with an information sheet explaining the study, was sent to all participating nurses via SMS using the hospital’s internal messaging system. The information sheet provided detailed explanations regarding the purpose and procedures of the study, ensuring participants could freely decide whether to participate. It was also emphasized that participants could withdraw at any time during the study without any repercussions. The survey was conducted anonymously, and no personal or institutional identifiers were collected. For in‐depth interviews, a co‐researcher affiliated with an external organization met the participants in person and provided detailed explanations of the study’s purpose before obtaining written consent. Gallup Korea’s professional researchers reviewed the consent forms before conducting the interviews. To facilitate participant reimbursement, Gallup Korea independently managed the consent forms. Interviews were audio‐recorded with the participants’ consent. After the interviews, the recordings were transcribed, and all recordings were destroyed following the completion of data analysis to ensure confidentiality and data security.

### 2.5. Analysis

#### 2.5.1. Quantitative Data Analysis

We analyzed the data using the SPSS Statistics program (version 26.0, IBM Corp., Armonk, NY). We examined differences in WPB experience (according to the participants’ general characteristics and work‐related aspects) using a *t*‐test and analysis of variance, and we scrutinized factors that differed significantly through a post hoc comparison using the Scheffe test. We investigated the correlations among pathological narcissism, perfectionism, dark personality, organizational culture, and WPB experience using Pearson’s correlation coefficients. We also analyzed the predictors of WPB experience via multiple regression analysis and tested the normality of residuals with the Kolmogorov–Smirnov test.

#### 2.5.2. Qualitative Data Analysis

We used Excel for coding as its cell‐based data reformulation aids narrative analysis and reduces hardware demands and errors, while filtering enhances quantification. We employed Colaizzi’s phenomenological method [32], which focuses on extracting common attributes among all participants. We repeatedly read the transcripts to holistically understand the participants’ experiences and gain insight into their feelings. Subsequently, we identified meaningful words and sentences directly related to the phenomenon to extract significant statements and then formulated them into general meanings. We integrated statements with similar meanings into themes and grouped similar themes into thematic clusters. We continuously verified whether the derived thematic clusters accurately reflected the participants’ expressions by reviewing them against the interview transcripts. An internal audit team (two researchers) reviewed the coding process and results to ensure that the procedures were applied systematically and transparently and to identify potential biases and inconsistencies.

To ensure the rigor of the qualitative research, we applied Guba and Lincoln’s [33] scientific research evaluation criteria. First, to enhance credibility, we transcribed the interview content verbatim and derived concepts using the participants’ own terminology, with a neutral attitude maintained during analysis of the results to avoid researcher bias. Second, for fittingness, two participants confirmed the consistency of the analyzed content with the participants’ descriptions of their experiences. Further, we received feedback on the appropriateness of the findings from a nursing professor with rich experience in conducting qualitative research. Third, we ensured auditability by providing a detailed description of the research method and analysis process as well as by directly quoting the participants’ words in the results; this allows the reader to verify our interpretation and analysis. Fourth, we established confirmability by adhering to credibility, fittingness, and auditability criteria. We have taken courses and lectures related to qualitative research and have vast experience in conducting qualitative research.

## 3. Results

### 3.1. Quantitative Data Analysis

#### 3.1.1. Participants’ General and Work‐Related Characteristics

The mean age of the participants was 29.57 years (standard deviation 5.20), while the mean total career length was 5.58 years (standard deviation 5.21). Of 161 participants, 144 (89.4%) were women and 126 (78.3%) were single. There were 145 staff nurses (90.1%), and 113 (70.2%) had completed WPB prevention education provided by their organization (Table 1).

**TABLE 1:** General and work‐related characteristics (*N* = 161).

Characteristics	Categories	*N* (%)	*M* (SD)
Age (years)			29.57 (5.20)

Sex	Female	144 (89.4)	
	Male	17 (10.6)	

Marital status	Single	126 (78.3)	
	Married	35 (21.7)	

Religion	No	97 (60.2)	
	Yes	64 (39.8)	

Educational level	3‐year college	3 (1.9)	
	Bachelor’s degree	144 (89.4)	
	≥ Master’s degree	14 (8.7)	

Total working years			5.58 (5.21)

Position	Staff nurse	145 (90.1)	
	Charge nurse	16 (9.9)	

Health status	Poor	82 (50.9)	
	Good	79 (49.1)	

WPBeducation	No	48 (29.8)	
	Yes	113 (70.2)	

*Note:* M: mean.

Abbreviation: SD, standard deviation.

#### 3.1.2. WPB Experience According to General and Work‐Related Characteristics

WPB experience did not significantly differ according to the general characteristics and work‐related characteristics, with the exception of subjective health status. Those with poor health status exhibited higher rates of WPB experience compared to those without poor health status (*t* = 2.33, *p* = 0.021) (Table 2).

**Table 2:** Comparison of WPB by general and work‐related characteristics (*N* = 161).

Characteristics	Categories	*N*	WPB (victim)		
			*M* (SD)	*t/F*	*p*
Sex	Female	144	48.30 (17.00)	1.43	0.156
	Male	17	42.12 (16.09)		

Marital status	Single	126	48.90 (16.91)	1.80	0.074
	Married	35	43.11 (16.61)		

Religion	No	97	47.88 (17.63)	0.21	0.833
	Yes	64	47.30 (16.03)		

Educational level	3‐year college	3	59.33 (9.02)	1.00	0.370
	Bachelor’s degree	144	47.74 (16.89)		
	≥ Master’s degree	14	44.21 (18.67)		

Position	Staff nurse	145	47.97 (16.89)	0.72	0.474
	≥ Charge nurse	16	44.75 (17.99)		

Health status	Poor	82	50.66 (17.93)	2.33	0.021
	Good	79	44.52 (15.40)		

WPB education	No	48	48.38 (16.75)	0.35	0.724
	Yes	113	47.34 (17.12)		

*Note:* M: mean.

Abbreviation: SD, standard deviation.

#### 3.1.3. Correlation Among Major Variables

WPB experience was positively correlated with pathological narcissism (*r* = 0.45, *p* < 0.001), perfectionism (*r* = 0.29, *p* < 0.001), and dark personality (*r* = 0.38, *p* < 0.001), while it was negatively correlated with positive organizational culture (*r* = −0.45, *p* < 0.001) (Table 3).

**Table 3:** Correlation among variables (*N* = 161).

	Pathological narcissism	Perfectionism	Dark personality	Positive organizational culture	WPB (victim)
	*r* (*p*)	*r* (*p*)	*r* (*p*)	*r* (*p*)	*r* (*p*)
Pathological narcissism	1				
Perfectionism	0.44 (< 0.001)	1			
Dark personality	0.69 (< 0.001)	0.43 (< 0.001)	1		
Positive organizational culture	−0.19 (0.018)	−0.21 (0.007)	−0.05 (0.529)	1	
WPB experience	0.45 (< 0.001)	0.29 (< 0.001)	0.38 (< 0.001)	−0.45 (< 0.001)	1

#### 3.1.4. Predictors of WPB Experience

Participant characteristics that showed significant differences in relation to WPB (marital status, health status) (*p* < 0.10) and variables identified as being correlated with WPB (pathological narcissism, perfectionism, dark personality, positive organizational culture) were entered into a regression model. There was no multicollinearity among the independent variables (VIF: 1.03–2.15), and independence of residuals was confirmed with a Durbin–Watson index of 1.91. The residuals also showed normal distribution (*Z* = 0.06, *p* = 279) and homoscedasticity (*χ* ^2^ = 2.79, *p* = 835). This regression model explained 35.4% of the variance of WPB experience. The most potent predictor of WPB experience was positive organizational culture (*β* = −0.39, *p* < 0.001), followed by pathological narcissism (*β* = 0.22, *p* = 0.022) and dark personality (*β* = 0.21, *p* = 0.025), that is, WPB experience increased with more negative organizational culture, more severe pathological narcissism, and more severe dark personality (Table 4).

**TABLE 4:** Factors influencing WPB experience (*N* = 161).

Variables	*B*	SE	*β* (*p)*	*t*	*p*	VIF	95% confidence interval	
							Lower	Upper
(Constant)	61.34	10.38		5.91	< 0.001		40.83	81.85
Marriage^†^	−4.92	2.64	−0.12	1.86	0.064	1.03	−10.13	0.30
Health status^†^	−1.58	2.24	−0.05	0.71	0.482	1.08	−5.99	2.84
Pathological narcissism	0.14	0.06	0.22	2.32	0.022	2.15	0.02	0.26
Perfectionism	0.01	0.09	0.01	0.07	0.943	1.35	−0.17	0.19
Dark personality	0.40	0.18	0.21	2.27	0.025	2.05	0.05	0.75
Positive organizational culture	−0.40	0.07	−0.39	5.79	< 0.001	1.11	−0.54	−0.27

*Note:* Adjusted *R* ^2^ = 0.354, *F* = 15.61, *p* < 0.001. Durbin–Watson’s *d* = 1.91 (du = 1.82, 4‐du = 2.18), Kolmogorov–Smirnov test (*Z* = 0.06, *p* = 0.279), Breusch–Pagan (*χ* ^2^ = 2.79, *p* = 0.835).

Abbreviations: SE, standard error; VIF, variance inflation factor.

^†^Dummy variable (reference): Marriage (single); health status (poor).

### 3.2. Qualitative Data Analysis

Among the 21 interview participants, 3 were male and 18 were female. Their ages ranged from 24 to 40 years, with a mean age of 29.90 ± 3.91 years. Fifteen participants were unmarried, and 13 had no religious affiliation. As for education level, two participants had an associate’s degree, 18 had a bachelor’s degree, and one had a master’s degree or higher. The average tenure in the current department was 2.84 ± 1.39 years, and total clinical experience was 5.10 ± 2.76 years.

In the qualitative analysis, we identified 798 significant statements, which we then integrated into 150 meaningful statements. From these, we derived seven themes representing the experiences of ICU nurses regarding WPB. We subsequently structured these themes into three thematic clusters with comprehensive meanings (Table 5). The derived thematic clusters were “discomfort between novice and experienced nurses,” “demand for strong sanctions against WPB,” and “appeal to improve human infrastructure and the work environment.”

**Table 5:** ICU nurses’ experiences with WPB.

Theme clusters	Themes
Discomfort between novice and experienced nurses	Novice nurses are forced to endure discriminatory practices
	Distancing from novice nurses

Demand for strong sanctions against WPB	Need for a stronger reporting and punishment system
	Separation of victims and perpetrators

Appeal to improve human infrastructure and the work environment	Urgent need for additional staff
	Creating a culture of mutual care and respect
	Demand for career development and fair compensation

#### 3.2.1. Discomfort Between Novice and Experienced Nurses

There were clear differences in perceptions and relational tensions between novice and experienced nurses across work performance and interpersonal interactions. Novice nurses perceived that hierarchical organizational cultures constrained their ability to freely express their opinions or clinical judgments, and this environment functioned as a persistent source of psychological burden during daily work. In contrast, experienced nurses reported feeling burdened by the reactions and work approaches of novice nurses, reflecting a gap in mutual understanding. These perceptual discrepancies extended beyond individual interpersonal conflicts and manifested as relational tensions in perceptions of fairness in work allocation, interpretations of and responses to errors, and everyday communication practices. In particular, implicit expectations regarding roles and responsibilities operated differently according to levels of clinical experience. Consequently, novice nurses perceived themselves as being structurally disadvantaged within the organization, whereas experienced nurses tended to view such perceptions as excessive.

##### 3.2.1.1. Novice Nurses Are Forced to Endure Discriminatory Practices

Novice nurses in the ICU often find themselves invariably assigned to patients with higher acuity as well as a greater workload. Despite frequently being unable to leave work on time and facing extensive overtime, they reported that they rarely receive assistance from experienced colleagues. Moreover, they experience discrimination not only in patient assignments but also in the responses to their mistakes. While experienced nurses are treated leniently when making errors, mistakes made by novice nurses are rigorously addressed. They also feel discriminated against when they are expected to rectify errors made by experienced nurses during previous shifts, yet they feel compelled to endure such unfairness.

Participant 11: *I feel it’s unfair that when a newer nurse is the off-going nurse and misses something, she’ll [work] overtime to finish all the tasks. We work shifts, so you know, sometimes we make mistakes. But if an experienced nurse is the off-going nurse and misses something, that definitely becomes my work.*

##### 3.2.1.2. Distancing From Novice and Experienced Nurses

Experienced nurses expressed reluctance to approach novice nurses for work‐related inquiries or questions, citing their unfavorable reactions and differing mindsets. Despite not being overly critical of the mistakes made by novice nurses, experienced nurses noted a tendency among novice nurses to report even minor issues as harassment. This atmosphere of hypersensitivity led experienced nurses to avoid interactions with their novice nurses, resulting in reluctance to engage in necessary work‐related discussions.

Participant 14: *The hardest part was feeling really cautious and holding back because I was worried that even my well-intentioned actions or words could be misperceived and become a topic of gossip.*

#### 3.2.2. Demand for Strong Sanctions Against WPB

Participants emphasized that effectively addressing WPB requires more than the nominal existence of institutional policies. They underscored the need for a comprehensive and enforceable system that ensures accountability throughout the entire response process from reporting and investigation to disciplinary action and post‐incident management. In particular, participants highlighted that the absence of meaningful consequences for perpetrators and the lack of protective measures for victims undermine trust in existing mechanisms. They stressed that a credible system must both impose tangible sanctions on perpetrators and actively protect victims, including by preventing continued exposure to those responsible, in order to genuinely deter WPB and foster a safe working environment for novice nurses.

##### 3.2.2.1. Need for a Stronger Reporting and Punishment System

The participants expressed frustration that, even when WPB is reported to the human rights center, it is not thoroughly investigated and no punitive measures are enforced. As a result, they perceived the current system as being ineffective. They appealed for stringent punishment of perpetrators to prevent the recurrence of WPB.

Participant 2: *Even at the human rights center, they just tell you to attend a few hours of training. It’s all a formality and nothing really gets resolved. In the end, no one is held accountable.*

##### 3.2.2.2. Separation of Victims From Perpetrators

The participants opined that it is necessary to immediately separate the victim and perpetrator when WPB occurs. Managers should adjust the work schedule to avoid direct encounters between the victim and perpetrator or transfer them to different units. However, the participants felt that the separation of victims and perpetrators is not effectively implemented in reality.

Participant 5: *What most hospitals seem to do is just send both the victim and perpetrator to float to separate them, but I don’t think that’s being properly executed.*

#### 3.2.3. Appeal to Improve Human Infrastructure and the Work Environment

The participants believed that the intense work environment—where experienced nurses are responsible for overseeing novice nurses amidst an excessive workload—exacerbates WPB. They appealed for an increase in nursing staff and dedicated educators to continuously back up and train new nurses until they become proficient in their duties. They stressed the importance of establishing a culture of empathy and respect within the nursing organization. To foster this culture, they suggested initiatives that promote harmony and camaraderie as well as targeted educational programs. Additionally, they stressed the urgent need for a system and work environment that adequately compensate and recognize the contributions of nurses based on their experience and foster their professional growth.

##### 3.2.3.1. Urgent Need for Additional Staff

The participants noted that the high workload inevitably leads to increased stress among nurses, with experienced nurses having to oversee new nurses in an already demanding setting. This situation often results in experienced nurses projecting their heightened emotions onto new nurses and being strict to prevent any mistakes. The participants suggested that the workload should be adjusted by supplementing staff to free up capacity to teach new nurses or by allocating more dedicated educators for new nurses.

Participant 20: *If the work environment improves, we’d have more leeway, and we could extend OT, which would allow for more learning from mistakes. I think that if the work environment got better, bullying would also decrease.*

Participant 11: *A key solution seems to be having surplus staff. It would be great to have enough staff to fully back up new nurses or nurses who are less competent. Currently, I have to take care of my own patients and also look after new nurses, so my workload is double of what it usually is.*

##### 3.2.3.2. Creating a Culture of Mutual Care and Respect

The participants observed that experienced nurses often fail to recall their own experiences from when they were less experienced, while novice nurses lack respect for experienced counterparts. They expressed a desire for an organizational culture characterized by mutual understanding, respect, assistance, and collaboration. Additionally, they hoped for opportunities to bridge the communication gap and restore relationships between novice and experienced nurses.

Participant 2: *It seems like we just can’t remember our humble beginnings. It would be nice to have some kind of educational program where we could switch roles and do a bit of role-playing*.

##### 3.2.3.3. Demand for Career Development and Fair Compensation

The participants noted that the high workload inevitably leads to increased stress among nurses, with experienced nurses having to oversee new nurses in an already demanding setting. This situation often results in experienced nurses projecting their heightened emotions onto new nurses and being strict to prevent any mistakes. The participants suggested that the workload should be adjusted by *supplementing* staff to free up capacity to teach new nurses or by allocating more dedicated educators for new nurses.

Participant 4: *There’s no difference in salary between me and a new nurse. Even charge nurses end up with no salary difference compared to new nurses if they only work day shifts. So why should I have to clean up after new nurses? This evokes negative feelings in me, and the feedback I give tends to be less positive. If hospitals were to cultivate nurses’ careers and create a chart according to their career development, specifying the level of behavior required for each rank and providing corresponding compensation or time off, then experienced nurses would accept their duties more reasonably.*

## 4. Discussion

This study aimed to determine the predictors of WPB among ICU nurses and provide evidence to develop strategies for addressing WPB. A mixed‐methods approach was employed, combining a quantitative analysis of personality traits and organizational culture with a qualitative exploration of nurses’ interpersonal challenges, WPB experiences, and suggestions for improvement.

In the quantitative investigation, multiple regression revealed that positive organizational culture is the most potent predictor of WPB. This result aligns with the theme clusters identified in the qualitative investigation, particularly nurses’ advocacy to expand mutual understanding and respect and their appeal to improve human infrastructure and the work environment**.** These findings highlight the critical role of organizational culture in predicting and addressing WPB.

The finding that positive organizational culture emerged as the most potent predictor of WPB has crucial implications when considering the cultural characteristics of Korean nursing organizations. Korea has a strong hierarchical culture rooted in Confucian philosophy, where social norms emphasizing collectivism and conformity influence patterns of WPB [34]. While hierarchy plays an important role in organizational order, stability, efficiency, and member motivation, excessive control and authoritarian hierarchy lead to negative workplace behaviors such as bullying, blame culture, and incivility through rigid thinking and closed‐mindedness [35, 36]. Particularly in Confucian cultural contexts such as Korea, cultural norms emphasizing collectivism and respect for hierarchy influence how experienced nurses treat novice nurses [37], which connects directly to the qualitative theme of “discomfort between novice and experienced nurses” identified in this study. A culture that values seniority and the tendency to avoid conflict may serve as mechanisms that condone or conceal WPB. In organizations where rigid roles and authority within the hierarchy are perceived as power, there is a possibility of impersonal treatment and physical and emotional harm toward novice nurses. In such scenarios, victims find it difficult to actively respond against perpetrators in superior positions.

A more concerning finding is that nurses with past WPB victimization experiences were more likely to become perpetrators [38], demonstrating a vicious cycle of intergenerational transmission of bullying. Given that WPB tends to occur through abuse of power by experienced nurses targeting novice nurses with insufficient practical experience [39], educational programs and systems are needed to support experienced nurses in developing proper leadership skills to prevent WPB from becoming normalized under the guise of educating novice nurses. Additionally, considering that WPB management systems and policies have traditionally focused on victims, organizational and policy support is needed for experienced nurses who are at risk of becoming perpetrators.

The next most potent predictors of WPB experience were personality traits, pathological narcissism, and dark personality. In other words, WPB experience increased with more pronounced pathological narcissism and dark personality. Qualitative results also supported this, with participants identifying personality traits—such as an inability to empathize with others’ perspectives and a tendency to use abusive language and behavior—as primary causes of WPB. These findings warrant interpretation within the unique environmental context of ICUs.

The ICU environment possesses distinct characteristics that specifically trigger narcissistic behaviors and WPB. ICU nurses are especially vulnerable given the complexities of their work environment, which involve high‐acuity patients, life‐support systems, and advanced medical devices [40]. Unlike general nursing settings, ICUs operate under a zero‐tolerance error culture where minor mistakes, such as miscalculating vasoactive drug dosages, can result in immediate patient death [41]. This creates constant hypervigilance fundamentally different from other clinical settings. For nurses with narcissistic vulnerability, this transforms routine feedback into threats to their competence, triggering defensive behaviors such as blaming colleagues or devaluing others to protect their fragile self‐esteem [42]. Additionally, the rapid decision‐making demands during emergencies may normalize authoritarian communication, providing cover for aggressive behaviors under the guise of “clinical efficiency” [41]. The intense emotional labor of repeated exposure to patient suffering and death further depletes mentalization capacity, particularly for those with pre‐existing narcissistic traits [17, 40]. When psychological resources are exhausted, their limited capacity for perspective‐taking diminishes, increasing both perpetration and victimization of WPB [38].

Narcissistic vulnerability is characterized by craving attention and suffering diminished self‐esteem when failing to receive expected recognition [42]. In the ICU, competence is treated as baseline expectation rather than praised achievement, while errors receive immediate attention. This creates a paradoxical situation: the environment systematically denies the ego‐reinforcement that narcissistically vulnerable nurses require, potentially triggering maladaptive interpersonal behaviors including WPB [38, 42].

The impact of these individual personality characteristics on peer dynamics merits particular attention. Previous research has shown that greater narcissistic grandiosity and vulnerability were associated with higher WPB perpetration scores [38]. Among dark triad traits, psychopathy and Machiavellianism are linked to hard manipulative tactics such as threats and coercion, while Machiavellianism and narcissism are associated with soft tactics such as compliments and humor [43]. Individuals high in Machiavellianism prefer covert control, exerting dominance through subtle means such as asking numerous questions while revealing little about themselves, or employing flattery [44]. Within Korea’s hierarchical and collectivistic nursing organizational culture, such manipulative behaviors may operate more covertly, disguised as “senior guidance” or “teamwork emphasis.” This directly connects to the qualitative theme of “discomfort between novice and experienced nurses.”

The finding that positive organizational culture emerged as the most potent protective factor against WPB suggests, conversely, that when organizational norms fail to mitigate the negative effects of individual personality traits, WPB may rapidly proliferate. Therefore, healthcare organizations with ICUsin Korea should provide educational opportunities for nurses to understand their own and others’ personality characteristics and maintain appropriate interpersonal relationships, with particular emphasis on implementing intervention programs to enhance mentalization capacity [17]. Additionally, systemic approaches are needed to create environments that ensure transparent communication, clear rules, and psychological safety, while enabling early detection and sanctioning of narcissistic or dark personality traits when they manifest as manipulative behaviors.

Korean nurses’ responses to WPB differ markedly from Western approaches. Although Korea has established formal legislation requiring employers to investigate workplace harassment without delay and prohibit retaliation, nurses’ help‐seeking behaviors remain constrained by workplace norms and relational risks [45]. Qualitative research on tae‐um—a culturally recognized form of WPB in Korean hospital settings—reveals that nurses frequently respond through endurance‐based coping rather than utilizing formal mechanisms, reflecting how silence and self‐protection become embedded strategies in hierarchical clinical environments [46]. Studies consistently demonstrate that organizational silence is associated with WPB among Korean hospital nurses, suggesting that even when policies exist, the perceived cost of speaking up keeps reporting channels underused [47]. Notably, our qualitative findings identified “demand for strong sanctions against WPB” as a major theme, indicating that participants perceived current measures as insufficient despite Korea’s antibullying legislation, thereby underscoring a persistent gap between policy intent and practical effectiveness.

In contrast, Western systems explicitly institutionalize voice through dedicated reporting infrastructures. England’s National Health Service’s Freedom to Speak Up framework provides multiple pathways including designated Guardians, with national policy emphasizing that those who speak up should be thanked and received feedback on resulting actions—functions intended to normalize reporting [48]. The Australian Health Practitioner Regulation Agency’s notification framework enables reporting beyond the workplace through mandatory and voluntary notification pathways, providing external escalation routes when internal resolution is ineffective or unsafe [49]. This cross‐cultural contrast highlights that while Korea has a formal system, it functions less robustly when organizational norms favor silence and conflict avoidance, whereas Western contexts more visibly operationalize formal structures supporting escalations both within and outside employing organizations. Therefore, addressing WPB in Korean ICU nursing organizations requires not only adopting Western‐style formal reporting systems but also implementing culturally adapted approaches such as strengthened anonymous reporting mechanisms, explicit whistleblower protection, leadership education to break organizational silence, and organizational culture improvement programs emphasizing mutual respect.

### 4.1. Limitations

Given the cross‐sectional design, the causal relationship among the variables cannot be confirmed. As all data were collected through self‐reported surveys, recall bias cannot be entirely ruled out. The study findings are also limited in generalizability, as the sample was restricted to nurses working in ICUs at a single tertiary hospital in South Korea. Future studies should include a broader sample to verify the relationships between variables. Although this study was conducted among ICU nurses, we did not include quantitative or qualitative data that could directly explain the relationship between ICU dynamics and WPB. Future studies should incorporate designs that can directly explore these ICU‐specific mechanisms. Despite these limitations, the mixed‐methods approach provided valuable insights into WPB, capturing the nuanced experiences of nurses and their needs for prevention and intervention. These findings offer a strong foundation for developing actionable strategies to address WPB in nursing settings.

## 5. Conclusion

This study demonstrated that WPB was more prevalent in workplaces with negative organizational culture, and among individuals with higher levels of pathological narcissism and dark personality traits. Since WPB often stems from individual tendencies to reject differences and the absence of a culture of mutual care and respect, addressing these issues requires concerted efforts at both the individual and organizational levels. In this context, it is essential to understand the challenges faced by both novice and experienced nurses, identify their specific needs for improvement, and implement targeted solutions. As the nurses desired, it is important to implement policies that include strict sanctions to eliminate WPB, secure adequate staffing, and improve working conditions to reduce nurses’ workloads.

## 6. Implications for Nursing Management

As suggested by the surveyed nurses, nursing administrators should implement tailored strategies such as role‐playing, group training sessions, team‐building activities, and workshops that promote mutual understanding and collaboration between experienced and novice nurses to improve their relationships and foster a positive organizational culture. While it is important to provide mental health counseling and peer support for WPB victims, strict enforcement of sanctions and regulations against bullying behaviors is critical in preventing recurrence of WPB. Excessive workloads and poor working conditions often hinder respect and collaboration between novice and experienced nurses. Nursing management should address these issues by recognizing and fairly compensating nurses based on their contributions and workloads. Additionally, career development systems and improved work environments are essential to support professional growth and satisfaction. Although awareness of WPB has grown at societal and institutional levels, some nursing managers remain hesitant or passive in addressing WPB among nurses. Therefore, leadership training and the development of WPB response manuals are necessary to equip managers with the tools needed to intervene effectively.

## Author Contributions

Eunhye Kim contributed to conceptualization, methodology, validation, investigation, resources, supervision, project administration, writing–original draft preparation, and writing–review and editing. Sun Joo Jang contributed to conceptualization, methodology, software, data curation, formal analysis, visualization, validation, writing–original draft preparation, and writing–review and editing. Yujeong Kim contributed to methodology, data curation, formal analysis, validation, writing–original draft preparation, and writing–review and editing. Haeyoung Lee contributed to conceptualization, methodology, data curation, validation, investigation, funding acquisition, supervision, project administration, writing–original draft preparation, and writing–review and editing.

## Funding

This work was supported by the Basic Science Research Program through the National Research Foundation of Korea (NRF), which is funded by the Ministry of Education (grant number 2021R1F1A1046718).

## Disclosure

All authors have read and agreed to the final version of the manuscript.

## Conflicts of Interest

The authors declare no conflicts of interest.

## Data Availability

The data that support the findings of this study are available upon request from the corresponding author. The data are not publicly available due to privacy or ethical restrictions.
